# Multiparametric MRI biomarkers in pediatric osteosarcoma: associations of ADC, necrosis, and tumor volume with histologic and clinical outcomes in a retrospective cohort study

**DOI:** 10.1007/s00247-026-06665-4

**Published:** 2026-05-23

**Authors:** Isabela Azevedo Nicodemos da Cruz, Laura Marie Fayad, Carla Renata Pacheco Donato Macedo, Paulo Tarso Kawakami Perez, Mariana Batista Rosa Pinto, Bruno Henrique de Azevedo, Angela Grassato de Carvalho, Henrique Manoel Lederman, Artur Rocha Corrêa Fernandes, Júlio Brandão Guimarães

**Affiliations:** 1 Department of Diagnostic Imaging, Federal University of São Paulo, 715 - Rua Napoleão de Barros, Hospital São Paulo, São Paulo, 04023-900 Brazil; 2 Department of Musculoskeletal Radiology, Fleury S.A. (Brazil), São Paulo, Brazil; 3 The Russell H. Morgan Department of Radiology and Radiological Science, Johns Hopkins Medicine, Baltimore, USA; 4 Department of Oncology, Support group for adolescents and children with cancer, São Paulo, Brazil

**Keywords:** Osteosarcoma, Magnetic resonance imaging, Diffusion-weighted imaging, Apparent diffusion coefficient, Pediatric oncology, Neoadjuvant chemotherapy

## Abstract

**Background:**

Assessment of response to neoadjuvant chemotherapy in pediatric osteosarcoma is challenging, as histologic evaluation of tumor necrosis is only available after surgical resection. Quantitative MRI parameters, including diffusion-weighted imaging (DWI)–derived apparent diffusion coefficient (ADC) values, tumor volume, and radiologic necrosis, may provide noninvasive information related to treatment response and outcomes.

**Objective:**

To evaluate quantitative MRI features before and after neoadjuvant chemotherapy in pediatric osteosarcoma and to investigate their associations with histologic response and clinical outcomes.

**Materials and methods:**

This retrospective single-center cohort study included 50 pediatric patients with histologically confirmed osteosarcoma (2014–2023) who underwent MRI with DWI at diagnosis and after neoadjuvant chemotherapy. Tumor volume, radiologic necrosis, and ADC parameters (minimum, mean, and maximum) were independently assessed by two musculoskeletal radiologists. Histologic response was graded using the Huvos system. Associations between imaging parameters and histologic response, survival outcomes, and relapse were evaluated using nonparametric and exploratory time-to-event analyses.

**Results:**

Fifty pediatric patients were included (mean age 13.2 years; 62% male), of whom 21% had metastatic disease at diagnosis. Pre-treatment ADC minimum differed between good and poor histologic responders (median 661.75 vs 851.0×10⁻⁶ mm^2^/s; *P*=0.047). Tumor volume variation showed evidence of association with Huvos grade (Spearman *ρ*=−0.402, *P*=0.004). Post-treatment tumor volume was associated with clinical outcome (*P*=0.005), and larger pre- and post-treatment volumes were observed in patients who relapsed. All ADC parameters increased after chemotherapy (*P*<0.01). Pre-treatment ADC minimum showed evidence of association with overall survival (*ρ*=−0.300, *P*=0.038), while post-treatment ADC minimum was associated with disease-free survival (*ρ*=−0.458, *P*=0.028). ROC analysis of pre-treatment ADC minimum yielded an AUC of 0.68 for histologic response.

**Conclusion:**

Quantitative MRI parameters demonstrate exploratory associations with histologic response and clinical outcomes in pediatric osteosarcoma, providing additional information when interpreted alongside conventional MRI features and clinical data.

**Supplementary Information:**

The online version contains supplementary material available at 10.1007/s00247-026-06665-4.

## Introduction

Osteosarcoma is the most common malignant primary bone tumor in the pediatric population, accounting for approximately 5% of all pediatric cancers and nearly 20% of primary bone malignancies overall, predominantly affecting patients between 10 years and 30 years of age [1, 2].

Despite advances in multimodal therapy, prognosis of osteosarcoma remains dependent on the extent of disease at presentation, with 5-year survival ranging from 76% in localized disease to about 24% in cases with distant metastases [3]*.* Accurate local and systemic staging is fundamental for treatment planning and prognosis in osteosarcoma and magnetic resonance imaging (MRI) of the primary tumor is a key component in this process [4, 5]. Conventional sequences provide excellent soft tissue contrast and anatomical information, which are essential for surgical planning and evaluation of disease extent [6, 7].

Current clinical guidelines recommend neoadjuvant chemotherapy (NAC) for all stages of high-grade osteosarcoma, followed by a post-chemotherapy MRI for restaging and assessing resectability. Histologic quantification of tumor necrosis following chemotherapy is the most reliable prognostic indicator in osteosarcoma and determines if limb-sparing surgery is a viable treatment option [2, 8, 9]. However, because this assessment is only available postoperatively, its utility for early treatment adaptation is limited. Current evaluations of NAC efficacy are based primarily on clinical signs and conventional imaging, which lack standardized, quantifiable parameters and often fail to accurately reflect treatment response [2].

In this context, advanced quantitative MRI techniques appear as potential biomarkers for assessing therapeutic response. Diffusion-weighted imaging (DWI), in particular, evaluates the random motion of water molecules within tissue, quantified by the apparent diffusion coefficient (ADC). Areas with restricted diffusion are associated with dense, viable tumor tissue, while regions of necrosis typically show higher diffusivity and elevated ADC values [6, 7, 9, 10]. Tumors in the bone marrow, such as osteosarcoma, may develop fibrosis, inflammatory cell infiltration and granulation tissue in response to neoadjuvant therapy, resulting in lower ADC values compared to purely necrotic tumor. Such changes may be better assessed by DWI than conventional MRI sequences [11].

Previous studies suggest that ADC metrics may help distinguish responders from non-responders [2, 6]. In children, however, the interpretation of DWI and ADC values is challenging due to the variable content of red and fat marrow in different ages and puberal status [12]. Therefore, reference values are not established, and interpretation should be cautious.

This study aims to evaluate quantitative MRI parameters—including tumor volume, necrosis, ossification, and ADC values—before and after neoadjuvant chemotherapy in pediatric osteosarcoma, and to investigate their correlation with histologic response and clinical outcomes.

## Materials and methods

### Study design

This is a retrospective, observational, single-center cohort study approved by the Institutional Review Board and complies with the ethical standards of the Declaration of Helsinki and national regulations. Informed consent was waived due to the retrospective nature of the study and anonymization of subjects’ data.

### Population

One-hundred-thirty-four pediatric patients under 18 years of age with histologically confirmed osteosarcoma were identified consecutively through institutional databases by a study coordinator. All patients were first diagnosed between January 2014 and September 2023. Inclusion criteria: age <18 years at diagnosis, tumor detected at diagnosis, availability of pre- and post-treatment MRI with DWI sequences, anatomopathological results of pre-surgical biopsy and post-surgical analysis, and clinical follow-up at the same institution. Exclusion criteria: prior treatment and/or prior bone tumor, incomplete imaging or suboptimal image quality, incomplete treatment or follow-up data. All included patients had documented clinical follow-up at our institution after completion of neoadjuvant chemotherapy. Follow-up duration varied according to year of diagnosis. All patients underwent two cycles of neoadjuvant therapy regimen before surgery with doxorubicin, cisplatin, and methotrexate. No a priori sample size calculation was performed.

### Imaging acquisition

MRI examinations were conducted using a 1.5-tesla MR scanner (Achieva Philips Healthcare, Netherlands) under a standardized imaging protocol. The protocol included multiplanar T1-weighted (repetition time (TR)=510 ms, echo time (TE)=19 ms, slice thickness (ST)=4 mm, interslice gap=1 mm, number of excitations (NEX)=1, flip angle=150°) and T2-weighted with fat suppression (T2-FS) (TR=6,600 ms, TE=66 ms, ST=4 mm, interslice gap=1 mm, NEX=1, flip angle=132°) and/or short-TI inversion recovery (STIR) (TR=3,780 ms, TE=91 ms, inversion time (TI)=220 ms, ST=4 mm, interslice gap=1 mm, NEX=1, flip angle=132°) sequences in axial, sagittal, and coronal orientations.

In addition, a post-contrast T1-weighted fat-saturated 3D fast-spin-echo variable-flip-angle (SPACE) sequence (TR=450 ms, TE=11 ms, ST=1 mm, interslice gap=0 mm, NEX=1, flip angle=120°) was acquired in the axial plane with multiplanar reconstructions.

Diffusion-weighted imaging (DWI) was acquired in the axial plane using a single-shot echo-planar imaging (EPI) spin-echo sequence (TR=2,500 ms, TE=80 ms, ST=5 mm, interslice gap=1 mm, NEX=1, flip angle=180°, EPI factor=88, gradient strength of 50 mT/m, matrix 256×256). Two different *b*-values were used for DWI (50 s/mm^2^ and 800 s/mm^2^), which enabled differentiation between tissue components with varying diffusion properties. ADC maps were generated automatically on the scanner using a monoexponential model based on the two acquired *b*-values (*b*=50 s/mm^2^ and *b*=800 s/mm^2^), using the low *b*-value image as the reference for ADC calculation.

### Image assessment

Two board-certified radiologists (7 years and 5 years of experience musculoskeletal radiology, respectively) independently reviewed all images. Before independent review, a brief calibration session was performed using three sample cases from the cohort and the predefined imaging criteria and measurement approach were reviewed, including ADC ROI placement, tumor volume measurement, radiologic necrosis, and peritumoral T2 hyperintensity; inter-observer agreement reflects independent readings. The readers were blind in regard to pathology results and clinical outcomes.

Conventional and diffusion-weighted sequences were assessed to measure tumor volume, ADC values, enhancement, and necrosis, as described below, and all variables were documented pre- and post-treatment. For descriptive analysis, we evaluated the tumor’s predominant signal intensity on T1-weighted and fluid-sensitive/T2-weighted sequences relative to skeletal muscle, categorized as hypointense, isointense, or hyperintense when this pattern involved more than 50% of the tumor. Tumor heterogeneity was visually graded as absent, <25%, 25–50%, or >50% of the total tumor volume. Tumor margins were categorized as well-defined, poorly defined, or mixed. Well-defined margins were defined as sharply demarcated tumor borders along most of the lesion circumference, poorly defined margins as indistinct or infiltrative borders, and mixed margins as the coexistence of well-defined and poorly defined borders within the same tumor. Peritumoral T2 hyperintensity was assessed on fluid-sensitive sequences and categorized as (0) absent; (1) mild/moderate, defined as T2 hyperintensity extending less than 50% of the maximal tumor circumference; or (2) extensive, defined as T2 hyperintensity involving 50% or more of the maximal tumor circumference or extending beyond the immediate peritumoral region.

ADC values were measured using freehand ROIs separately in two tumor regions: osseous (tumoral component within cortical bone limits) and softtissue (extracortical tumoral component extending beyond cortical margins). ADC measurements were obtained on a single axial slice demonstrating the most representative solid tumor component, identified on anatomical MRI sequences and co-registered to the ADC map. ROIs were placed on the solid-appearing tumor portion on ADC maps with reference to anatomic and post-contrast images, prioritizing regions with the most restricted diffusion and/or enhancement when present. Areas of macroscopic cystic or necrotic change were excluded. For post-chemotherapy MRI, ROIs were placed using the same strategy, targeting the residual solid component (enhancing when present) and avoiding non-viable, necrotic tumor fluid collections; when extensive post-treatment heterogeneity was present, ROIs were placed in the dominant residual solid component identifiable on conventional and contrast-enhanced sequences. ADC minimum, mean, and maximum values were recorded. Additionally, reference ADC measurements were obtained from normal marrow regions known to undergo early fatty conversion to confirm technical consistency of ADC acquisition and provide internal quality control across examinations. This was used to identify markedly inconsistent ADC values that could suggest ADC map acquisition or calculation errors; these measurements were not included in outcome analyses, as they were not study endpoints. Representative examples of ROI placement are provided in figure cases.

Variables analyzed:

- Tumor location: Bone where the lesion is centered.
- Tumor volume: Quantitative variable measured in milliliters (mL) based on manual measurement in three axes from MRI images, calculated using the formula of an ellipsoid mass volume=[(π/6)×height×width×depth] [13]. Total tumor volume included both intraosseous and extraosseous components. The extraosseous softtissue component was measured separately by identifying the tumor portion extending beyond the cortical bone margins. Measurements were obtained using multiplanar assessment (axial, coronal, and sagittal planes) on fluid-sensitive and post-contrast sequences to accurately delineate the extraosseous tumor boundaries. When the extraosseous component was circumferential and wrapped around the involved bone, the volume of the intraosseous component was subtracted from the total tumor volume to estimate the extraosseous softtissue volume. The same ellipsoid formula was applied to this extraosseous component. Representative examples of tumor volume measurement are provided in Supplemental Fig. 1. Tumor volume measurements were performed by a single radiologist using the predefined measurement protocol to ensure internal consistency across cases.
- Apparent diffusion coefficient (ADC): Minimum, mean, and maximum ADC values were extracted from the osseous component tumor, as well as mean ADC values of soft tissue component. Values were expressed in mm^2^/s×10⁻⁶. For all statistical analyses, including group comparisons and ROC analyses, the average ADC value from the two independent reviewers was used as the representative measurement for each parameter. ADCmin, ADCmean, and ADCmax were measured exclusively in the osseous tumor component (within cortical bone limits), while ADCsoft referred to the ADCmean measured in the extraosseous softtissue component.
- ΔADC values: Representing the absolute variation in ADC values between pre- and post-treatment measurements, calculated separately for ADCmin, ADCmean, and ADCmax.
- %ΔADC values: Representing the relative variation (percentage) in ADC values between pre- and post-treatment measurements, calculated separately for ADCmin, ADCmean, and ADCmax.
- Enhancement: Defined as visually estimated enhancing viable tumor on post-contrast fat-saturated T1-weighted images, expressed as the percentage of enhancing tissue relative to the total tumor volume. Axial and reconstructed multiplanar post-contrast images were reviewed. Categorical variable based on radiological characteristics, divided into four groups: (0) absent, (1) <25%, (2) 25–50%, and (3) >50%. The average enhancement grade from the two independent reviewers was used for statistical analyses.
- Necrosis: Defined as visually estimated nonenhancing necrotic tissue relative to the total tumor volume. Necrotic areas were characterized by irregular margins, T1 hypointensity, T2 hyperintensity, and absence of post-contrast enhancement. Necrosis was assessed using multiplanar review of fluid-sensitive sequences and post-contrast fat-saturated T1-weighted images, including axial acquisitions and multiplanar reconstruction. Categorical variable based on radiological characteristics, graded as: (0) absent, (1) <25%, (2) 25–50%, and (3) >50%. Change in imaging necrosis grade was defined as the difference between post- and pre-treatment grades. An increase corresponded to a higher post-treatment grade, a decrease to a lower post-treatment grade, and no change to identical grades before and after NAC. For statistical analysis, the average necrosis grade of the two independent reviewers was used as the representative value for each examination.

**Fig. 1:**
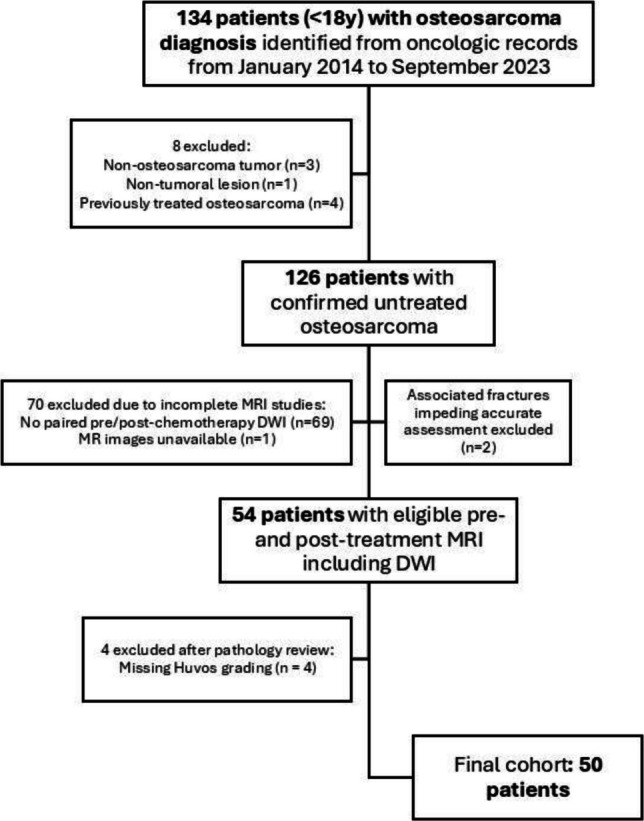
Flowchart depicting patient selection, with exclusion criteria applied and final cohort. From 134 pediatric patients with histologically confirmed osteosarcoma initially identified, exclusions were applied, yielding a final cohort of 50 patients

### Clinical data

Clinical data were retrieved from electronic medical records by a study coordinator and reviewed by the study investigators. Extracted data included age, sex, clinical stage (localized or metastatic), surgical records, chemotherapy records, and outcomes. Patients were followed from diagnosis to death or last consultation, until September 2024.

Variables analyzed:

- Metastasis at diagnosis: Presence and anatomical sites of metastases were recorded. Metastatic locations (pulmonary, skeletal, lymphatic, and others) were not mutually exclusive, and patients could present with more than one site. For statistical analyses, metastasis at diagnosis was primarily evaluated as a binary variable (presence vs absence of distant metastasis), while specific metastatic sites were described descriptively.
- Time-to-treatment interval: Time interval between date of diagnosis and date of first chemotherapy
- Time-to-surgery interval: Time interval between date of diagnosis and date of surgery
- Overall survival: Time from date of diagnosis to death from any cause or last follow-up, whichever occurs first. Patients alive at last follow-up were censored at the date of last clinical follow-up.
- Disease-free survival: Time from date of diagnosis to first documented relapse. Patients without relapse at last follow-up were censored at the date of last clinical follow-up.
- Relapse location: Presence and anatomical sites of relapse were recorded. Relapse locations (local recurrence, nodal, pulmonary, distant bone, and medullary) were not mutually exclusive. For statistical purposes, relapse was analyzed as a binary outcome (yes/no), while specific relapse sites were described descriptively. Recurrences were confirmed primarily by histopathological analysis. In selected cases where biopsy was not performed, recurrence was determined based on imaging progression on MRI in conjunction with clinical and multidisciplinary team assessment.
- Progression interval: Time interval between date of treatment start (first chemotherapy) and date of relapse
- Outcome status: Final patient status categorized as (1) alive without disease, (2) alive with disease, (3) deceased due to cancer, (4) deceased due to other causes, and (0) missing data.

### Histopathology

All biopsy and resection specimens were analyzed by pediatric pathologists. Histologic subtypes were recorded (osteoblastic, chondroblastic, telangiectatic, etc.). Tumors were graded by nuclear atypia, mitotic activity, and cellularity determined by pathologic inspection of biopsy material obtained prior to chemotherapy. Chemotherapy response was assessed via the Huvos system and patients with >90% tumor necrosis were considered good responders, whereas patients with ≤90% tumor necrosis were considered poor responders.

Variables analyzed:

- Histologic subtype: Tumors were categorized into defined histopathological types, including osteoblastic, chondroblastic, telangiectatic, epithelioid, fusocellular, undefined, or other, according to biopsy and surgical specimen review.
- Histologic grade: Pre-treatment histologic grade was categorized as: (1) low grade, (2) intermediate grade, or (3) high grade.
- Huvos response: The degree of tumor necrosis following chemotherapy, classified as: Grade I (0 to ≤49% necrosis), Grade II (50 to ≤89% necrosis), Grade III (90 to ≤99% necrosis), Grade IV (100% necrosis). For statistical analysis, histologic response was binarized to good response (Grades III and IV) and poor response (Grades I and II).

### Statistical analysis

Statistical analyses were conducted using R software version 4.4.2, adopting a statistical significance level of 5% (*P*<0.05).

Descriptive statistics summarized demographic, clinical, and imaging data, including presentation of means, standard deviations, medians, interquartile ranges, frequencies, and percentages. Inter-observer agreement was assessed separately for continuous and categorical MRI variables. Continuous variables, including ADC parameters (ADCmin, ADCmean, ADCmax, and ADCsoft), were evaluated using intraclass correlation coefficients (ICC). Tumor volume measurements were obtained by a single reader and therefore were not subjected to inter-observer analysis. Categorical variables, including necrosis grade, tumor heterogeneity, predominant T1-weighted and fluid-sensitive/T2-weighted signal, tumor margins, and presence of peritumoral T2 hyperintensity, were assessed using Cohen’s kappa statistics.

Comparisons between independent groups were performed using the Mann–Whitney test for continuous variables and the chi-square test for categorical variables. When appropriate, the Kruskal–Wallis test was applied for comparisons involving more than two groups. The association between continuous variables was assessed using Spearman’s correlation coefficient. To investigate the discriminatory ability of continuous variables in relation to binary outcomes, receiver operating characteristic (ROC) curve analyses were conducted, with calculation of the area under the curve (AUC) and corresponding confidence intervals. ROC analyses were performed for multiple clinical endpoints, including cancer-related death, relapse, alive without disease status, and poor histologic response according to Huvos classification. Exploratory Cox regression analyses were performed for time-to-event outcomes (relapse and death). Due to the limited number of outcome events, multivariable Cox regression models were restricted to adjustment for a limited number of clinically relevant covariates (age at diagnosis and metastatic status) to reduce the risk of model overfitting. Given the exploratory nature of this study and the relatively small sample size, primary analyses were initially performed without formal correction for multiple comparisons. As a sensitivity analysis, false discovery rate (FDR) correction was subsequently applied to the multiple pairwise association analyses presented in Tables 4, 5, and 6. Results should therefore be interpreted as hypothesis-generating.

## Results

### Patient demographics and clinical-histological analyses

Of the 134 patients identified with osteosarcoma diagnosis, 84 were excluded due to incomplete MRI studies (*n*=70), non-osteosarcoma lesion (*n*=4), previously treated osteosarcoma (*n*=4), incomplete pathology data (*n*=4), and fracture impeding accurate assessment (*n*=2). A total of 50 pediatric patients diagnosed with osteosarcoma were included in this study after exclusion criteria were applied (Fig. 1). The mean age at diagnosis was 13.2 years (range, 6–18 years; median, 14.0 years), and 62% (31/50) were male. Demographic, clinical, and histopathological characteristics of the cohort are summarized in Table 1.

**Table 1:** Demographic, clinical, and histopathological characteristics of the study cohort

Variable	*N*/total (%) or value
Total patients	50
Age at diagnosis, years	
Mean (range)	13.2 (6–18)
Median	14.0
Sex	
Male	31/50 (62%)
Female	19/50 (38%)
Tumor location	
Femur	20/50 (40%)
Tibia	17/50 (34%)
Humerus	9/50 (18%)
Fibula	2/50 (4%)
Pelvis	1/50 (2%)
Rib	1/50 (2%)
Clinical stage at diagnosis	
Localized disease	37/47 (79%)
Metastatic disease	10/47 (21%)
Missing staging data	3/50 (6%)
Metastatic site at diagnosis^a^	
Pulmonary	7/47 (15%)
Bone	2/47 (4%)
Other	1/47 (2%)
Histologic subtype	
Osteoblastic	21/50 (42%)
Chondroblastic	12/50 (24%)
Telangiectatic	3/50 (6%)
Fusocellular	2/50 (4%)
Other/undefined	12/50 (24%)
Histologic grade	
Intermediate grade	3/50 (6%)
High grade	47/50 (94%)
Clinical outcome at last follow-up	
Alive without disease	14/46 (30%)
Alive with disease	19/46 (41%)
Deceased due to cancer	13/46 (28%)
Missing outcome data	4/50 (8%)
**Follow-up**	
Mean follow-up time	47 months

ᵃMetastatic sites were not mutually exclusive; patients could present with more than one metastatic site

Age showed no evidence of association with outcome status (*P*=0.137) or disease-free survival (*P*=0.165), but was associated with overall survival (*P*=0.027). Sex was not associated with outcome status (*P*=0.572) or overall survival (*P*=0.519).

The most frequent tumor sites were femur 40% (20/50), tibia 34% (17/50), humerus 18% (9/50), fibula 4% (2/50), pelvis 2% (1/50), and rib 2% (1/50). There was no significant association between tumor location and outcome status (*χ*^2^ *P*=0.539), disease-free survival (*P*=0.834), or overall survival (*P*=0.167).

Histologically, the osteoblastic subtype was most common (42% [21/50]), followed by chondroblastic (24% [12/50]). Other subtypes included telangiectatic (*n*=3) and fusocellular (*n*=2). There was no significant association between histologic subtype and outcome status (*P*=0.089), disease-free survival (*P*=0.108), or overall survival (*P*=0.535).

High-grade tumors accounted for 94% (47/50), and intermediate grade for 6% (3/50). Pre-treatment histologic grade was significantly associated with outcome status (*P*=0.007) and patients with grade 1 tumors had the highest proportion of disease-free survivors (50%). Mean overall survival decreased with increasing grade (*P*=0.014).

Staging information at diagnosis was available for 47 of 50 patients. Among these, 79% (37/47) had localized disease, and 21% (10/47) had distant metastasis (pulmonary 15% [7/47], bone 4% [2/47], others 2% [1/47]). Among patients with metastatic disease, only 2 were alive and disease-free at last follow-up, compared to 12 in the non-metastatic group. Metastasis at diagnosis showed evidence of association with disease-free survival (*P*=0.016) and overall survival (*P*=0.020).

Nearly half of the patients (46%−23/50) were classified as Huvos grade 1, and 24% (12/50) achieved grade 4 (complete necrosis). Grades 2 and 3 were observed in 16% (8/50) and 8% (4/50) of cases, respectively. Patients with grades 3 and 4 necrosis had the highest proportions of disease-free survivors and no cancer-related deaths, whereas poor responders (1 and 2) were associated with higher mortality. This association with outcome status was statistically significant (*P*=0.004). Mean disease-free survival increased with Huvos grade: 472.7 days for grade 1, 784.4 days for grade 2, 1,165.6 days for grade 3, and 1,275.2 days for grade 4 (*P*=0.008). A similar trend was observed for overall survival: 1,310.1 days for grade 1, 1,726.7 days for grade 2, 1,963.7 days for grade 3, and 2,027.4 days for grade 4 (*P*=0.023).

### Treatment timeline and outcomes

The mean interval between diagnosis and initiation of chemotherapy (*time-to-treatment*) was 46.4 days (SD=66.7), and surgery occurred at a mean of 123.6 days after diagnosis (SD=36.0).

Clinical outcome data were available for 46 of 50 patients. Among these, 14 patients (30%) were alive without evidence of disease, 19 (41%) were alive with persistent or recurrent disease, and 13 (28%) had died from osteosarcoma at last follow-up. Outcome data were unavailable for 4 patients due to incomplete records.

Among patients who experienced disease recurrence, the disease-free survival averaged 627.6 days (median=458.0; range, 112.0–2,694.0 days), and the progression interval averaged 575.6 days (SD=523.2). The most common site of recurrence was pulmonary metastasis, observed in 17 patients. Local recurrence and regional lymph node involvement were each observed in 2 patients, while distant bone and medullary involvement were observed in 4 patients each. Some patients presented recurrence in more than one anatomical site.

The overall survival had a mean of 1,485.2 days and a median of 1,396.0 days. Mean follow-up time was 47 months.

### Imaging

Overall, high inter-observer agreement was observed for categorical variables, including DWI signal, enhancement grade, necrosis grade (kappa range 0.72–1.00). For continuous variables, including tumor volume and ADC measurements, inter-observer agreement was excellent (ICC range 0.73–0.92).

**Fig. 2:**
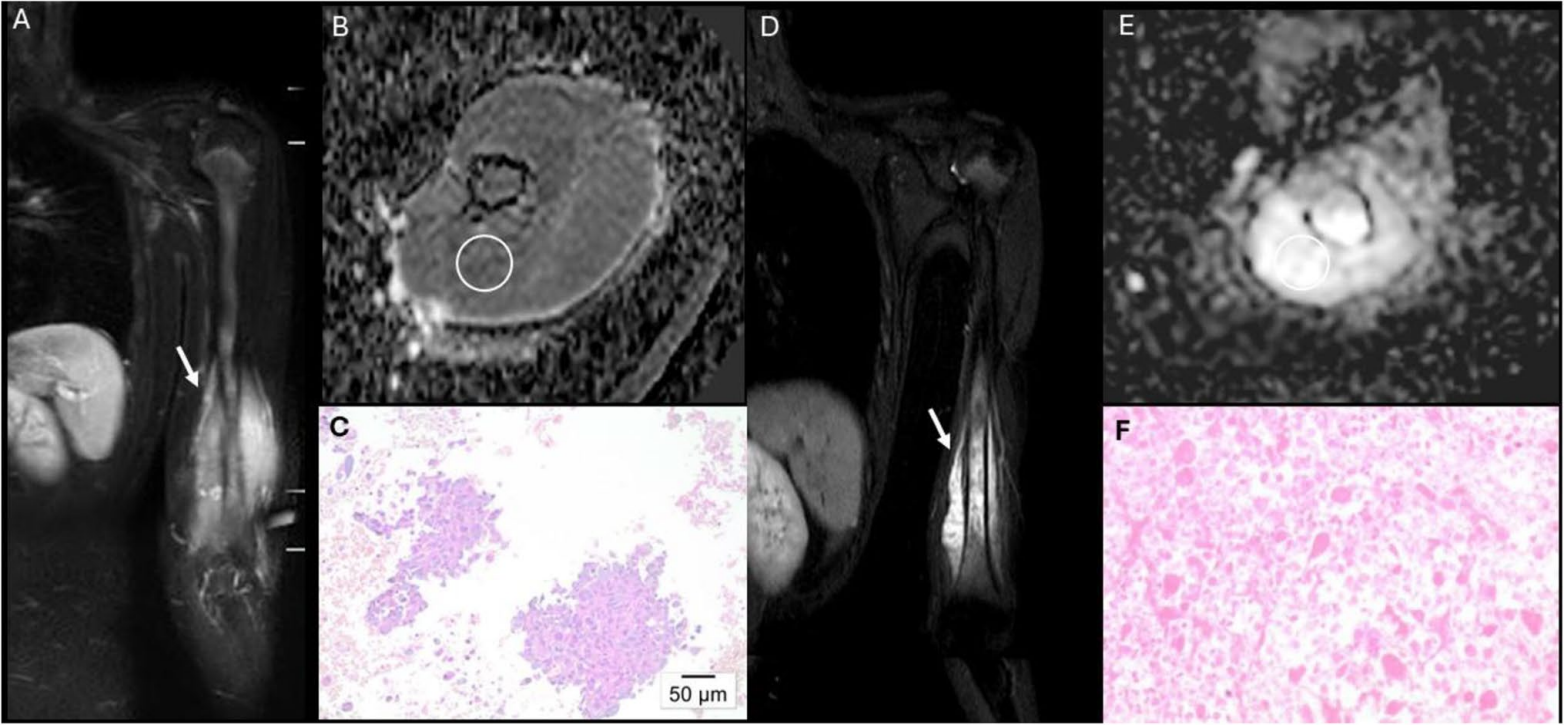
14-year-old male with osteosarcoma of distal humerus. Pre-treatment MRI demonstrates a large tumor with high signal intensity on coronal STIR image (*arrow* in **A**). The ADCmin measured 900×10⁻⁶ mm^2^/s within the osseous tumor component (**B**). Histopathological analysis (**C**) demonstrated viable tumor tissue with high-grade neoplastic cells. After neoadjuvant chemotherapy, MRI shows reduction of the softtissue component on coronal STIR image (*arrow* in **D**). The ADCmin increased to 2,200×10⁻⁶ mm^2^/s in the corresponding region (**E**). Histopathological examination (**F**) demonstrated extensive necrosis without viable tumor cells (Huvos grade IV)

**Fig. 3:**
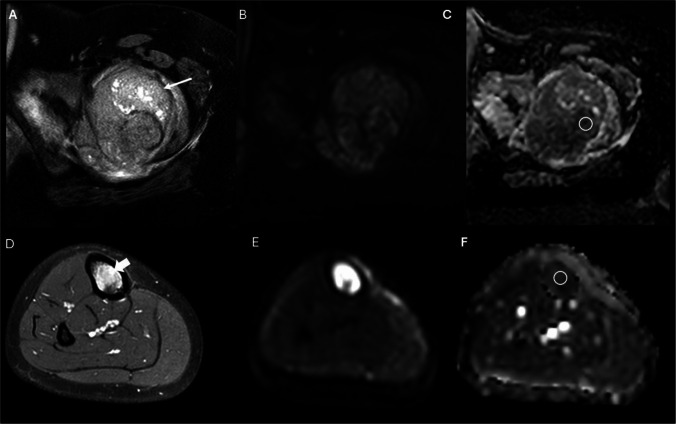
Representative examples illustrating differences in pre-treatment ADCmin between good and poor histologic responders. 14-year-old male with osteosarcoma of the proximal femur and good histologic response after neoadjuvant chemotherapy (Huvos grade III). Pre-treatment axial contrast-enhanced T1-weighted fat-suppressed image (**A**) demonstrates the solid enhancing osseous tumor with soft tissue component (*arrow*). Axial diffusion-weighted image (**B**) shows corresponding high signal intensity, and the ADC map (**C**) demonstrates low ADC within the osseous tumor component, with ADCmin measuring 672.5×10⁻⁶ mm^2^/s. The patient developed pulmonary relapse and was alive with disease at last follow-up. A 15-year-old male with osteosarcoma of the tibia shaft and poor histologic response after neoadjuvant chemotherapy (Huvos grade I). Pre-treatment axial contrast-enhanced T1-weighted fat-suppressed image (**D**) demonstrates the solid enhancing osseous tumor component (*arrowhead*). Axial diffusion-weighted image (**E**) shows corresponding diffusion signal abnormality, and the ADC map (**F**) demonstrates higher ADC within the osseous tumor component compared with the good responder, with ADCmin measuring 1,273.0×10⁻⁶ mm^2^/s. The patient developed pulmonary relapse and died from the disease

**Fig. 4:**
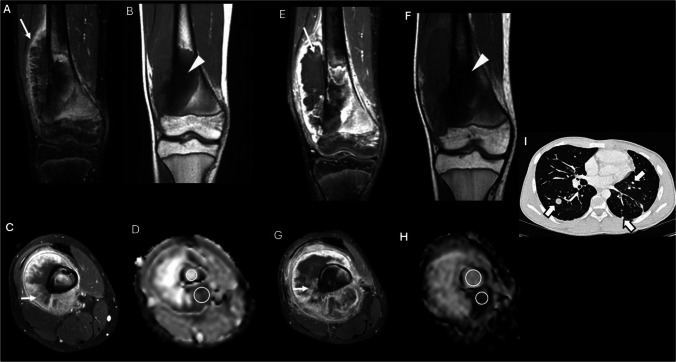
14-year-old male with osteosarcoma of the distal femur. Pre-treatment MRI (**A**-**D**) demonstrates a bone tumor with a large area of internal necrosis within the softtissue component on contrast-enhanced T1 fat-suppressed images (*arrows* in **A** and **C**) and partial ossification within the osseous component on T1-weighted images (*arrowhead* in **B**). The mean ADC measured 1,280×10⁻⁶ mm^2^/s in the osseous component and 1,350×10⁻⁶ mm^2^/s in the softtissue component (**D**). After neoadjuvant chemotherapy (**E**-**H**), MRI demonstrates increased and extensive necrosis with peripheral enhancement on contrast-enhanced T1 fat-suppressed images (*arrows* in **E** and **G**), as well as increased ossification on T1-weighted images (*arrowhead* in **F**). The mean ADC measured 1,300×10⁻⁶ mm^2^/s in the osseous component and 1,450×10⁻⁶ mm^2^/s in the softtissue component (**H**). Histopathology demonstrated a Huvos grade I response. Six months after completion of treatment, the patient developed pulmonary metastases (*thick arrows* in **I**) and died from the disease 1 year after relapse

**Fig. 5:**
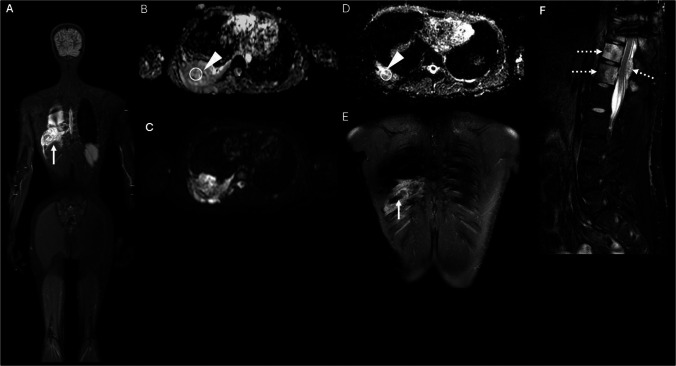
13-year-old male with osteosarcoma of the left rib. Pre-treatment MRI demonstrates a large mass arising from the left ninth rib with a prominent softtissue component on T2 fat-suppressed whole-body imaging (*arrow* in **A**). The mean ADC within the osseous component measured 1,300×10⁻⁶ mm^2^/s (*arrowhead* in **B**; **C** shows the axial DWI image). After neoadjuvant chemotherapy, MRI demonstrates a marked decrease in tumor volume and intralesional ossification on coronal T2 fat-suppressed images (*arrow* in **E**). The mean ADC increased to 1,880×10⁻⁶ mm^2^/s (*arrowhead* in **D**). Histopathology demonstrated a Huvos grade II response. Two years after surgery, the patient developed locoregional relapse involving adjacent vertebral bodies, with a softtissue component contributing to vertebral canal stenosis (*dotted arrows* in **F**), and subsequently died from the disease

On baseline MRI, the tumors were predominantly isointense to muscle on T1-weighted images and hyperintense T2-weighted images (*n*=47, 94%, for both). Baseline tumor heterogeneity was <25% in 38% (19/50), 25–50% in 21% (11/50), >50% in 10% (5/50), and absent/homogeneous in 31% (15/50). Margins were well-defined in 48% (24/50), mixed in 44% (22/50), and poorly defined in 8% (4/50). Baseline peritumoral T2 hyperintensity was mild/moderate in 70% (35/50), extensive in 24% (12/50), and absent in 6% (3/50). Figures 2, 3, 4, and 5 and Supplemental Figs. 1 and 2 illustrate cases.

### Tumor volume

Mean pre-treatment total tumor volume was 469.7 mL, and post-treatment volume was 408.7 mL, resulting in a mean variation of −61.5 mL. A volume increase in total tumor volume occurred in 48% (24/50) of patients.

The mean volume of soft tissue component before chemotherapy was 264.9 mL, and the mean volume after treatment was 216.8 mL, resulting in a mean variation of −53.0 mL. Twenty patients (41.7%) experienced an increase in soft tissue component volume after chemotherapy. Three patients demonstrated marked post-treatment volumetric increases (+443 mL to +834 mL), with poor histologic response (Huvos I–II), and imaging review indicated that the increase was predominantly attributable to expansion of intratumoral necrotic and hemorrhagic component rather than solid enhancing tumor growth.

There was no significant difference in pre- or post-treatment mean total tumor volume between good and poor responders (*P*>0.05). Total tumor volume variation demonstrated a significant negative correlation with Huvos grade (*ρ*=−0.402, *P*=0.004), and a similar correlation was observed for the softtissue component (*ρ*=−0.509, *P*=0.003).

When clinical outcome was analyzed in three groups (alive without disease, alive with disease, deceased), baseline total tumor volume did not differ significantly between groups (*P*=0.473). Softtissue volume variation, defined as the post-minus pre-NAC extraosseous tumor volume, differed significantly across outcome categories (*P*=0.036), whereas total tumor volume variation did not (*P*=0.078).

In comparisons between patients with and without relapse, baseline total tumor volume did not differ significantly (mean, 612 mL vs 338 mL; *P*=0.120). However, baseline softtissue tumor volume was significantly higher in patients who developed relapse (mean, 1681 mL vs 301 mL; *P*=0.012). Neither total tumor volume variation (*P*=0.187) nor softtissue volume variation (*P*=0.062) was significantly associated with relapse.

In survival analysis using Cox regression, total tumor volume was not associated with time to death (HR 1.76; *P*=0.116), nor was baseline softtissue volume (HR 0.97; *P*=0.743). Softtissue volume variation demonstrated a borderline association with time to death (HR 1.78; *P*=0.050), whereas total tumor volume variation was not associated with mortality (*P*=0.651).

When tumor volume variation was dichotomized, 24 patients (48%) demonstrated an increase in total tumor volume and 26 patients (52%) demonstrated volume reduction. Both total and extraosseous tumor volume demonstrated significant associations with final patient status (*P*=0.035 for both). Among those with total tumor volume reduction, 40% were disease-free, versus only 19% in the group with volume increase. Similarly, 41% of patients with extraosseous volume reduction were disease-free, versus 14% among those with increased extraosseous volume.

### MRI enhancement and necrosis

The proportion of tumors with moderate to extensive necrosis (grades 2–3) increased from 54% (27/50) to 78% (39/50) after chemotherapy (*P*=0.012; Supplementary Table S1). Inter-observer agreement for imaging necrosis grade was excellent before NAC (weighted kappa=0.91; 95% CI, 0.81–1.00) and substantial after NAC (weighted kappa=0.77; 95% CI, 0.62–0.92). At baseline, 54% (27/50) of tumors were classified as grades 2–3 necrosis. There was no significant association between imaging necrosis grade (pre-NAC, post-NAC), nor between change in necrosis grade from pre- to post-NAC, and histologic response when analyzed as good responders (Huvos III–IV) versus poor responders (Huvos I–II) (*P*=0.071).

Among the 50 patients, imaging necrosis grade increased after NAC in 18 patients (36%), remained unchanged in 22 patients (44%), and decreased in 10 patients (20%). Among patients with increased necrosis grade, 47% were alive and disease-free at last follow-up, compared with 20% in the no-increase group (stable or decreased necrosis). However, this difference did not reach statistical significance (*P*=0.095).

Mean disease-free survival (DFS) was 677.6 days in the increase group vs 632.6 days in the no-increase group (*P*=0.585) and mean OS was 1,541.7 days in the increase group vs 1,535.6 days in the no-increase group (*P*=0.972).

### ADC analysis – diffusion-weighted MRI

Across the cohort, all three parameters (ADCmin, ADCmean, and ADCmax) showed a significant average increase following chemotherapy (*P*<0.01). All metrics demonstrated good or excellent inter-observer agreement (Table 2).

**Table 2:** ADC metrics^a^ pre- and post-chemotherapy with inter-observer correlation (ICC)

	Measure	ADCmin^b^	ADCmean^b^	ADCmax^b^	ADC soft tissue^b^
Inter-observer agreement (R1 vs R2)	Pre-treatment	0.79	0.84	0.73	0.80
	Post-treatment	0.91	0.92	0.90	0.84
Average of R1 and R2	Pre-treatment	870.7	1,284.3	1,721.3	1,386.5
	Post-treatment	1,270.0	1,682.3	2,045.1	1,800.0
	ΔADC	+399.3	+398.1	+323.8	401.5
	%ΔADC	+79.4	+44.0	+31.2	+59.8
*N*=50	ADC increase	80%	72%	76%	69%
*N*=50	ADC decrease	20%	28%	24%	24%

^a^ADC values and ΔADC values are expressed as ×10⁻⁶ mm^2^/s. %ΔADC values are expressed as percentages. ^b^*ADCmin*, minimum ADC; *ADCmean*, mean ADC; *ADCmax*, maximum ADC; *ADC soft tissue*, mean ADC of soft tissue component; *ΔADC*, absolute ADC variation; *%ΔADC*, relative ADC variation

#### Correlation of ADC and Huvos grade

Due to the small number of patients in certain Huvos categories (particularly grades II and III), analyses were performed using a binary classification (good vs poor responders). Pre-treatment ADCmin differed between response groups (median 851.0×10⁻⁶ mm^2^/s in poor responders vs 661.75×10⁻⁶ mm^2^/s in good responders; *P*=0.047). Other pre- and post-NAC ADC parameters, as well as absolute and percentage changes in those parameters, did not achieve statistical significance (Table 3).

**Table 3:** ADC metrics^a^ pre- and post-chemotherapy and histological response

Variable	*P-*value	Poor response^c^ (median)	Good response^c^ (median)
Pre-treatment ADCmin^b^	**0.047**	851.0	661.75
Pre-treatment ADCmean^b^	0.097	1,249.5	1,018.25
Pre-treatment ADCmax^b^	0.466	1,758.0	1,567.0
Pre-treatment ADCsoft^b^	0.061	1,127.8	1,312.0
Post-treatment ADCmin^b^	0.205	1,091.5	986.5
Post-treatment ADCmean^b^	0.452	1,659.5	1,411.75
Post-treatment ADCmax^b^	0.762	2,193.5	1,780.0
Post-treatment ADCsoft^b^	0.076	1,856.8	1,530.0
ΔADCmin^b^	0.982	336.0	177.75
ΔADCmean^b^	0.955	463.0	278.5
ΔADCmax^b^	0.955	313.5	284.75
ΔADCsoft^b^	0.715	507.5	279.25
%ΔADCmin^b^	0.806	38.5	15.9
%ΔADCmean^b^	0.946	51.4	26.3
%ΔADCmax^b^	0.928	22.6	23.9
%ΔADCsoft^b^	0.910	36.5	26.5

^a^ADC values and ΔADC values are expressed as ×10⁻⁶ mm^2^/s. %ΔADC values are expressed as percentages. ^b^*ADCmin*, minimum ADC, *ADCmean*, mean ADC; *ADCmax*, maximum ADC; *ADC soft tissue*, mean ADC of soft tissue component; *ΔADC*, absolute ADC variation; *%ΔADC*, relative ADC variation. ^c^Poor response=Huvos 1 or 2; Good response=Huvos 3 or 4

**Fig. 6:**
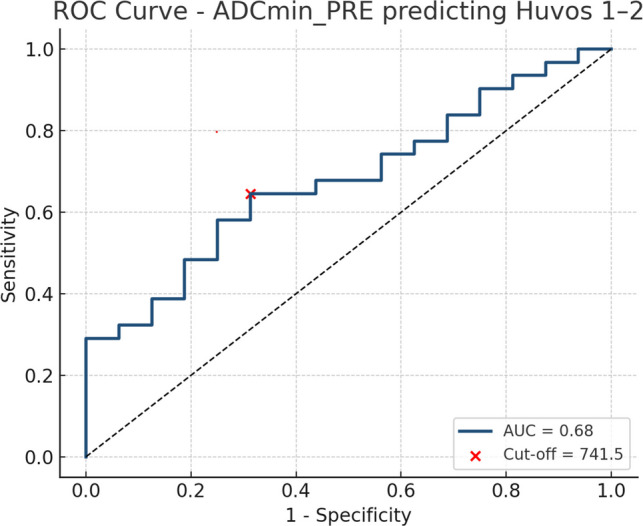
Receiveroperating characteristic (ROC) analysis of pre-treatment minimum apparent diffusion coefficient (ADCmin) for discrimination of poor histologic responders (Huvos I–II) vs good responders (Huvos III–IV) shows modest performance with AUC (area under the curve)=0.68. The optimal threshold by Youden’s index (741.5×10⁻⁶ mm^2^/s) yielded sensitivity of 65% and specificity of 69%

ROC analysis for pre-treatment ADCmin yielded an AUC of 0.68 (95% CI, 0.51–0.85), indicating modest discriminatory performance. The Youden-derived cut-off (741.5×10⁻⁶ mm^2^/s) provided 65% sensitivity and 69% specificity (Fig. 6).

#### Correlation of ADC and clinical features

Patients with metastatic disease at diagnosis demonstrated higher baseline ADC values compared with patients with localized disease (Supplementary Table S2). Baseline ADC of the softtissue component was significantly higher in patients with metastatic disease (median 2,152×10⁻⁶ mm^2^/s vs 1,168×10⁻⁶ mm^2^/s; *P*=0.028). Treatment-related ADC changes also differed between groups. Patients with localized disease demonstrated greater increases in ADC values following chemotherapy, with statistically significant differences for ΔADCsoft (*P*=0.018), %ΔADCmean (*P*=0.036), and %ΔADCsoft (*P*=0.018) after FDR correction (Supplementary Table S2). Spearman correlation analysis showed no significant associations between age at diagnosis and baseline ADC parameters or treatment-related ADC changes (all *P*>0.05), with weak correlation coefficients (|*ρ*|≤0.18).

Analysis according to primary tumor site was not feasible because of the small number of cases in several anatomical categories, which precluded reliable statistical comparison. Similarly, association between ADC parameters and histologic grade could not be meaningfully evaluated because most tumors were high grade (47/50), with only three cases of intermediate grade.

#### Correlation of ADC with clinical outcomes

Comparison across three clinical outcome status groups (alive without disease, alive with disease, deceased) demonstrated differences for pre-treatment ADCmin (*P*=0.025) and ADCsoft (*P*=0.016) (Table 4). Reported group medians were calculated from the averaged reader values for each ADC parameter. Median ADCmin was lowest in the alive without disease group (650×10⁻⁶ mm^2^/s) and highest in the deceased group (986×10⁻⁶ mm^2^/s). The alive without disease group showed lower median values of ADCsoft. No other ADC parameters differed significantly across groups.

**Table 4:** Comparison of pre-treatment ADC values and treatment-related ADC changes^a^ (absolute and percentage) across clinical outcome status groups (Kruskal–Wallis test)

Variable	Alive without disease (*N*=14)		Alive with disease (*N*=19)		Deceased due to cancer (*N*=13)		*P-*value
	Mean (S.D.)	Median [Q1;Q3]	Mean (S.D.)	Median [Q1;Q3]	Mean (S.D.)	Median [Q1;Q3]	
Pre-treatment ADCmin^b^	**619 (221)**	**650 [541;717]**	**930 (424)**	**851 [617;1,188]**	**1,099 (591)**	**986 [641;1,534]**	**0.025**
Pre-treatment ADCmean^b^	1,027 (262)	1,000 [831;1,266]	1,412 (481)	1,438 [1,024;1,732]	1,446 (667)	1,409 [970;2,024]	0.053
Pre-treatment ADCmax^b^	1,465 (407)	1,412 [1,122;1,804]	1,897 (545)	1,882 [1,502;2,315]	1,824 (799)	1,620 [1,168;2,646]	0.094
Pre-treatment ADCsoft^b^	**1,064 (390)**	**1,078 [740;1,168]**	**1,536 (487)**	**1,312 [1,186;1,982]**	**1,620 (742)**	**1,642 [1,000;2,228]**	**0.016**
ΔADCmin^b^	401 (452)	254 [110;456]	288 (497)	85.0 [13.8;789]	401 (837)	270 [−251.50;669]	0.507
ΔADCmean^b^	450 (457)	296 [93.5;728]	287 (626)	463 [−154.75;804]	371 (890)	298 [−84.00;642]	0.783
ΔADCmax^b^	460 (625)	285 [47.8;681]	163 (836)	238 [−494.25;592]	314 (1,029)	235 [47.5;636]	0.768
ΔADCsoft^b^	643 (661)	641 [243;941]	261 (628)	218 [−7.00;558]	285 (1,079)	484 [−484.50;842]	0.370
%ΔADCmin^b^	60.3 (79.5)	35.0 [13.4;68.4]	72.5 (187)	14.3 [1.43;61.5]	100 (198)	33.8 [−16.42;99.6]	0.584
%ΔADCmean^b^	51.6 (55.8)	33.2 [7.79;88.4]	29.4 (51.3)	31.3 [−10.94;76.2]	45.1 (80.6)	26.1 [−10.34;77.8]	0.531
%ΔADCmax^b^	40.1 (62.7)	23.9 [2.44;43.7]	16.6 (52.5)	10.4 [−23.53;43.2]	36.3 (74.0)	15.0 [2.29;74.7]	0.598
%ΔADCsoft^b^	89.7 (101)	65.9 [22.5;131]	21.1 (42.0)	19.2 [−1.24;43.8]	76.8 (176)	24.1 [−21.01;59.3]	0.175

^a^ADC values and ΔADC values are expressed as ×10⁻⁶ mm^2^/s. %ΔADC values are expressed as percentages. ^b^*ADCmin*, minimum ADC; *ADCmean*, mean ADC; *ADCmax*, maximum ADC; *ADC soft tissue*, mean ADC of soft tissue component; *ΔADC*, absolute ADC variation; *%ΔADC*, relative ADC variation

In comparisons between patients with and without relapse, ΔADCmean differed between groups in unadjusted analysis (Table 5). No significant differences in ADC parameters were observed between survivors and patients who died in univariate comparisons (all *P*>0.05). Although higher median baseline ADCmin and ADCsoft values were observed in patients who died, these differences did not reach statistical significance (Table 6).

**Table 5:** Comparison of pre-treatment ADC values and treatment-related ADC changes^a^ (absolute and percentage) across patients with and without relapse (Mann-Whitney test)

Variable	No relapse (*N*=26)		Relapse (*N*=24)		*P-*value
	Mean(S.D.)	Median [Q1;Q3]	Mean (S.D.)	Median [Q1;Q3]	
ADCmin^b^	762 (411)	674 [603;892]	989 (469)	885 [602;1,343]	0.081
ADCmean^b^	1,192 (455)	1,104 [890;1,319]	1,384 (551)	1,423 [949;1,750]	0.125
ADCmax^b^	1,644 (535)	1,631 [1,210;1,845]	1,804 (680)	1,865 [1,208;2,294]	0.313
ADCSoft^b^	1,263 (456)	1,148 [969;1,434]	1,515 (677)	1,444 [1,126;2,110]	0.097
ΔADCmin^b^	504 (449)	370 [132;904]	286 (709)	97.5 [−152.12;509]	0.060
ΔADCmean^b^	**546 (434)**	**643 [214;874]**	**238 (801)**	**168 [−324.62;590]**	**0.043**
ΔADCmax^b^	500 (617)	425 [155;715]	133 (954)	139 [−430.50;540]	0.062
ΔADCsoft^b^	571 (560)	498 [190;987]	232 (959)	138 [−488.50;786]	0.116
%ΔADCmin^b^	92.0 (163)	38.5 [15.9;100]	69.6 (155)	14.0 [−15.19;82.1]	0.144
%ΔADCmean^b^	52.1 (43.1)	53.4 [12.7;84.4]	35.2 (75.1)	11.1 [−16.61;77.0]	0.116
%ΔADCmax^b^	37.6 (54.3)	24.8 [8.64;45.4]	24.3 (66.6)	6.81 [−18.52;70.6]	0.162
%ΔADCsoft^b^	56.1 (64.4)	46.3 [15.2;96.1]	63.6 (148)	9.60 [−23.32;62.0]	0.191

^a^ADC values and ΔADC values are expressed as ×10⁻⁶ mm^2^/s. %ΔADC values are expressed as percentages. *ADCmin*, minimum ADC; *ADCmean*, mean ADC; *ADCmax*, maximum ADC; *ADCsoft*, mean ADC of soft tissue component; *ΔADC*, absolute ADC variation; *%ΔADC*, relative ADC variation

**Table 6:** Comparison of pre-treatment ADC values and treatment-related ADC changes^a^ (absolute and percentage) between patients who remained alive and those deceased (Mann–Whitney test)

Variable	Alive (*N*=37)		Deceased (*N*=13)		*P-*value
	Mean(S.D.)	Median [Q1;Q3]	Mean (S.D.)	Median [Q1;Q3]	
ADCmin^b^	790 (366)	729 [599;936]	1,099 (591)	986 [641;1,534]	0.071
ADCmean^b^	1,227 (436)	1,151 [870;1,440]	1,446 (667)	1,409 [970;2,024]	0.269
ADCmax^b^	1,685 (534)	1,718 [1,220;1,997]	1,824 (799)	1,620 [1,168;2,646]	0.699
ADCsoft^b^	1,302 (499)	1,186 [962;1,617]	1,620 (742)	1,642 [1,000;2,228]	0.089
ΔADCmin^b^	399 (494)	246 [82.0;900]	401 (837)	270 [−251.50;669]	0.732
ΔADCmean^b^	408 (557)	463 [−4.00;844]	371 (890)	298 [−84.00;642]	0.514
ΔADCmax^b^	327 (734)	312 [1.00;596]	314 (1,029)	235 [47.5;636]	0.782
ΔADCsoft^b^	448 (667)	375 [138;981]	285 (1,079)	484 [−484.50;842]	0.617
%ΔADCmin^b^	74.0 (143)	34.4 [9.34;82.3]	100 (198)	33.8 [−16.42;99.6]	0.946
%ΔADCmean^b^	43.7 (53.2)	43.6 [−0.46;80.4]	45.1 (80.6)	26.1 [−10.34;77.8]	0.650
%ΔADCmax^b^	29.4 (55.7)	23.4 [0.09;45.6]	36.3 (74.0)	15.0 [2.29;74.7]	0.903
%ΔADCsoft^b^	53.1 (78.5)	31.0 [6.73;89.4]	76.8 (176)	24.1 [−21.01;59.3]	0.583

^a^ADC values and ΔADC values are expressed as ×10⁻⁶ mm^2^/s. %ΔADC values are expressed as percentages. ^b^*ADCmin*, minimum ADC; *ADCmean*, mean ADC; *ADCmax*, maximum ADC; *ADCsoft*, mean ADC of soft tissue component; *ΔADC*, absolute ADC variation; *%ΔADC*, relative ADC variation

Because multiple exploratory comparisons were performed across ADC parameters, false discovery rate (FDR) correction was applied as a sensitivity analysis to Tables 4, 5, and 6; after adjustment, none of these group-comparison findings remained statistically significant.

Univariable Cox regression analysis demonstrated an association between pre-treatment ADCmin and time to death (HR=1.16; 95% CI, 1.03–1.30; *P*=0.013). No other ADC parameters showed significant associations with time-to-event outcomes in univariable Cox analyses.

Additional multivariable Cox regression analyses adjusted for age at diagnosis and metastatic status were performed to account for potential clinical confounding. After adjustment, no ADC parameters remained significantly associated with either time to relapse or time to death (Supplementary Tables S3 and S4).

Spearman correlation analysis was performed to evaluate associations between ADC variables and progression interval. Pre-treatment ADCmin demonstrated a negative correlation with progression interval (*ρ*=−0.35, *P*<0.05; *n*=46). No other significant correlations were observed.

For prediction of cancer-related death, AUC values ranged from 0.452 to 0.561. All corresponding 95% confidence intervals included 0.5, indicating no evidence of discriminatory performance above chance level. For alive without disease and poor histologic response, AUC values ranged from 0.435 to 0.647 and from 0.465 to 0.569, respectively, with all confidence intervals including 0.5.

For relapse prediction, AUC values ranged from 0.635 to 0.667. ΔADCmean demonstrated the highest performance (AUC=0.667; 95% CI, 0.509–0.825). This confidence interval excluded 0.5, indicating modest discrimination in this cohort (Table S5). The optimal cut-off for ΔADCmean was 421.25×10⁻⁶ mm^2^/s, yielding sensitivity of 0.67, specificity of 0.65, positive predictive value of 0.64, and negative predictive value of 0.68 (Table S5).

## Discussion

In this study, pre-treatment ADCmin showed evidence of association with histologic response and with time to death in exploratory survival analysis. ADC-based metrics also showed associations with clinical outcome status and relapse in unadjusted analysis. Overall, these findings suggest that quantitative diffusion metrics may provide exploratory signals relevant to risk stratification in pediatric osteosarcoma.

DWI is a well-established advanced technique in oncology and ADC measurements provide quantitative data on tumor cellularity and treatment response in many neoplastic diseases, such as soft tissue sarcomas and multiple myeloma [5, 14–16]. However, correlation of ADC and histological and clinical outcomes in osteosarcoma remains controversial in the literature. Due to the intralesional heterogeneity of osteosarcomas, particularly after chemotherapy, mean ADC values may not reliably represent the most cellular tumor components [17, 18].

ADC minimum has been proposed to better reflect areas of high cellularity and therefore to be more valuable for assessing histologic response [14, 17]. In our cohort, pre-treatment ADCmin was associated with histologic response and with time to death in univariable analysis, with modest discriminatory power for histologic response (AUC 0.68). Associations with broader clinical outcome groupings were observed in unadjusted analyses, suggesting potential as a prognostic biomarker. In addition, ADCmin showed the greatest relative change between pre- and post-chemotherapy studies, consistent with the literature [2, 18].

ADCmean is commonly used in clinical practice due to its reproducibility, reflecting overall tissue composition, while ADCmax represents low cellularity regions of the tumor [5]. In our analysis, ΔADCmean differed between patients with and without relapse and ROC analysis demonstrated modest discriminatory performance, suggesting potential prognostic value.

All ADC metrics demonstrated significant post-treatment increases, consistent with the biological effects of chemotherapy, with a change from densely cellular tumor to necrotic or disorganized tissue with greater water mobility and support the concept that ADC progression reflects treatment-related changes [9, 17, 19–21].

In our cohort, variation in ADC parameters was not consistently associated with histologic response. Prior studies have reported heterogeneous findings, often limited by small sample sizes and methodological variability [17, 19, 20, 22–28]. In combination with our findings, the available evidence suggests that although no established cut-off exists, ADC changes may provide complementary information regarding treatment response [7, 9].

In our cohort, increases in ADC were not always paralleled by favorable histologic response, reflecting the complex interplay of multiple factors such as tumor matrix composition, calcification, hemorrhage, fibrosis, and necrosis. In particular, matrix-rich subtypes such as chondroblastic osteosarcoma may demonstrate intrinsically higher baseline ADC values, independent of treatment effect. Because ADC measurements were obtained using ROI-based sampling rather than whole-lesion volumetric analysis, subtle intratumoral heterogeneity may not have been fully captured. Therefore, ADC variation is best understood when considered in conjunction with morphological characteristics and the clinical context.

Radiologic necrosis increased after chemotherapy, although it did not correlate with Huvos response. Patients with increasing necrosis tended to remain disease-free longer and had higher overall survival, suggesting a possible contribution to clinical decision-making.

In our cohort, tumor volume reduction after NAC correlated with better histologic response, in accordance with earlier studies [13]. The baseline softtissue tumor component was significantly higher in patients who developed relapse and softtissue volume variation differed across clinical outcome groups and showed a borderline association with time to death. However, total tumor volume was not significantly associated with relapse or survival outcomes and variation in total volume was not associated with recurrence. These findings partially align with previous studies suggesting linking greater tumor burden to poorer prognosis [19, 29, 30].

Although tumor baseline size and shrinkage are known prognostic markers in various malignancies, interpretation must be cautious in osteosarcoma. The tumor’s dense and mineralized matrix often persists after chemotherapy, which may lead to minimal reduction even in good responders, and expansion of the necrotic or hemorrhagic component may increase tumor size [6, 30, 31].

Our study has several limitations. Although the cohort included 50 patients, subgroup analyses reduced statistical power and limited the feasibility of multivariable modeling. Established prognostic factors, such as metastatic status and tumor grade, may have acted as confounders, and the number of outcome events constrained adjustment for these variables. Multiple exploratory analyses were performed, increasing the risk of type I error. ADC measurements were obtained from single-slice ROIs and therefore may not fully represent whole-tumor heterogeneity. In addition, ROI placement was subjective, and although inter-observer agreement for ADC measurements was assessed, variability specifically attributable to ROI placement was not separately evaluated. Finally, although imaging acquisition followed a standardized protocol, minor variations across the long study interval cannot be excluded. These findings should therefore be interpreted as exploratory and hypothesis-generating, pending validation in larger prospective cohorts.

Still, our study provided valuable information on the correlation of ADC with clinical outcomes, beyond histological response, which has been the focus of most previous investigations. When interpreted alongside conventional MRI features and clinical data, quantitative MRI parameters—particularly ADC metrics—provide meaningful information for both clinical and histologic outcomes in pediatric osteosarcoma and may improve preoperative assessment and risk stratification.

## Supplementary Information

Below is the link to the electronic supplementary material.

ESM 1 (DOCS 724 KB)

ESM 2 (DOCS 46.4 KB)

## Data Availability

Data supporting the findings of this study are available from the corresponding author upon reasonable request.
