# Supplementary material for: Multiparametric MRI biomarkers in pediatric osteosarcoma: associations of ADC, necrosis, and tumor volume with histologic and clinical outcomes in a retrospective cohort study
Source: Pediatr Radiol. 2026 May 23;56(7):1572–87. doi: 10.1007/s00247-026-06665-4 (PMC13357396; doi:10.1007/s00247-026-06665-4)
Supplement: Supplementary file 1 — (DOCS 724 KB) [file 247_2026_6665_MOESM1_ESM.docx]

SUPPLEMENTARY INFORMATION

**
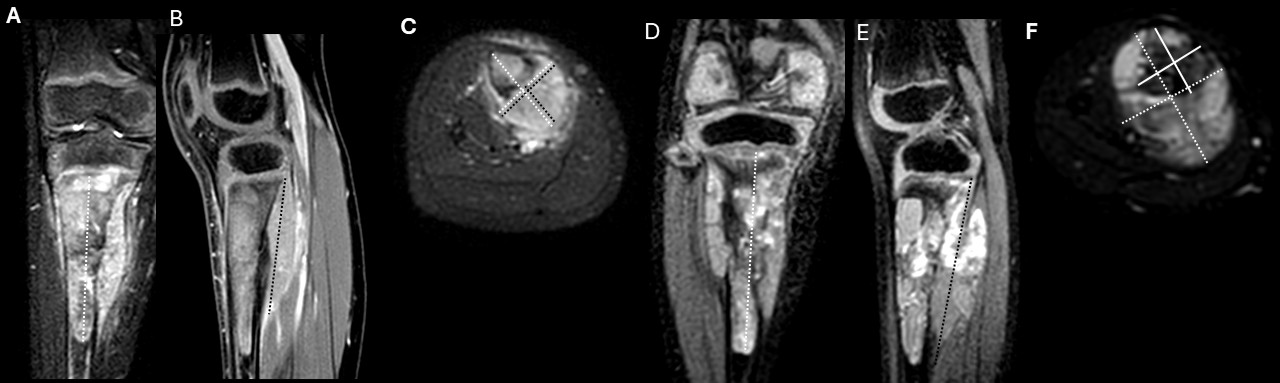
**

**Supp Figure 1.** 7-year-old male with osteosarcoma of the proximal tibia. Pre-treatment contrast-enhanced T1-weighted fat-suppressed MR images in the coronal (A), sagittal (B), and axial (C) planes demonstrate a large heterogeneously enhancing tumor with an extraosseous soft-tissue component. Total tumor volume was estimated using the maximal tumor dimensions in three orthogonal planes (white dotted lines). The extraosseous soft-tissue component was measured separately using the maximal dimensions of the extracortical tumor component (black dotted lines). Post-treatment contrast-enhanced T1-weighted fat-suppressed MR images in the coronal (D), sagittal (E), and axial (F) planes demonstrate an increase in both total tumor volume and the extraosseous soft-tissue component. Post-treatment total tumor volume was estimated from maximal tumor dimensions in three orthogonal planes (white dotted lines). Because the extraosseous component was nearly circumferential and partially surrounded the involved bone, the extraosseous soft-tissue volume was estimated by subtracting the intraosseous tumor volume from the total tumor volume. The longitudinal dimension of the extraosseous component is illustrated on the sagittal image (black dotted line in E), while the axial image (F) shows the total tumor cross-section (white dotted line) and the intraosseous component used for subtraction (white solid line).

**
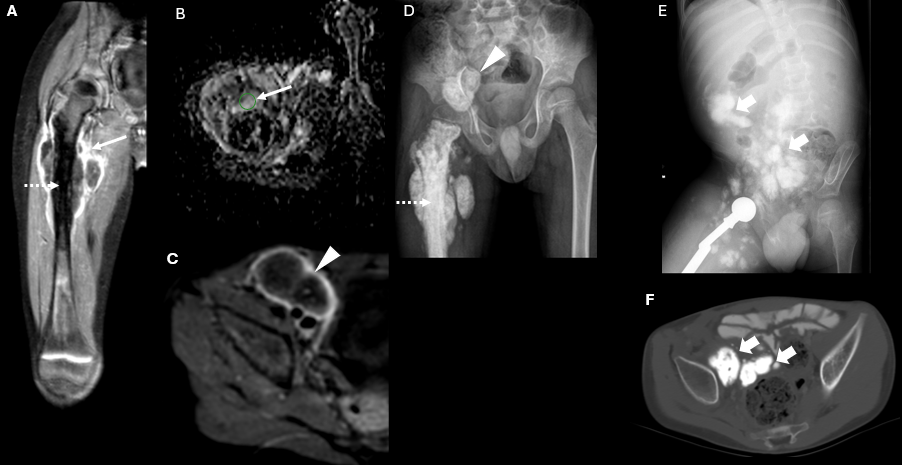
**

**
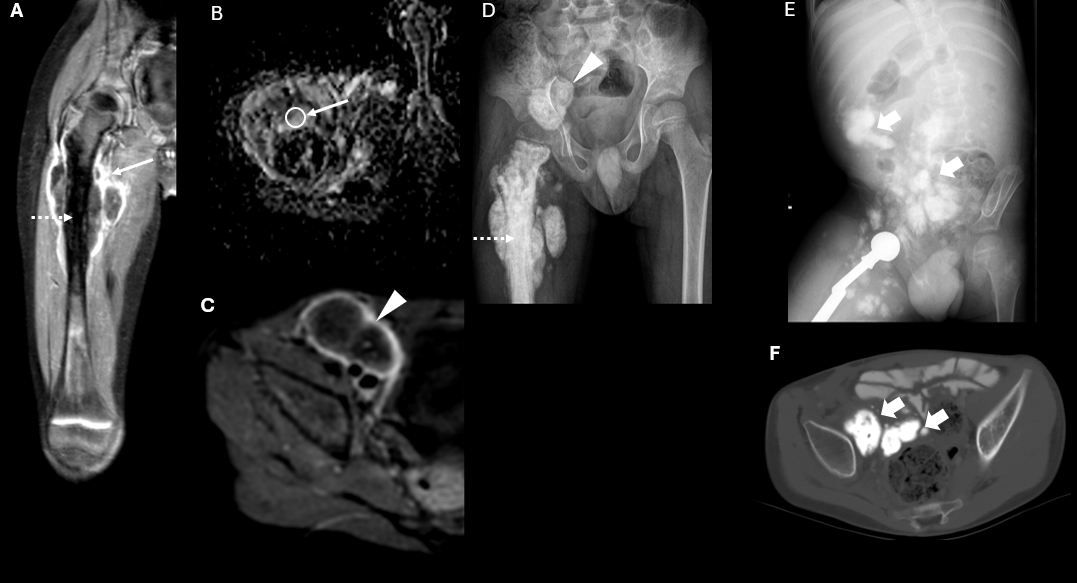
**

**Supplemental Figure 2. 4-year-old male with osteosarcoma of the proximal femur.** Pre-treatment MRI (A–C) demonstrates a large tumor with peripheral enhancement on contrast-enhanced T1 fat-suppressed images (arrow in A) and intralesional ossification (dotted arrow), also visible on the conventional radiograph (D). The mean ADC measured 1600 ×10⁻⁶ mm²/s within the soft-tissue tumor component (arrow in B). A large ossified nodal metastasis was identified in the inguinal lymph node chain (arrowhead in C and D). The patient underwent limb-sparing surgery and later developed additional ossified nodal masses with extensive peritoneal involvement (thick arrows in E (conventional radiograph) and F (CT) and obstructive hydronephrosis (not shown), and died one year later from the disease.
