# Supplementary material for: Multiparametric MRI biomarkers in pediatric osteosarcoma: associations of ADC, necrosis, and tumor volume with histologic and clinical outcomes in a retrospective cohort study
Source: Pediatr Radiol. 2026 May 23;56(7):1572–87. doi: 10.1007/s00247-026-06665-4 (PMC13357396; doi:10.1007/s00247-026-06665-4)
Supplement: Supplementary file 2 — (DOCS 46.4 KB) [file 247_2026_6665_MOESM2_ESM.docx]

**SUPPLEMENTARY TABLES**

**Table S1.** Tumor Necrosis Pre- and Post-Chemotherapy with Interobserver Agreement (Weighted Kappa)

|  | Pre-chemotherapy | | | Post-chemotherapy | | |
| --- | --- | --- | --- | --- | --- | --- |
|  | R1 | R2 | Mean | R1 | R2 | Mean |
| Grade 0^a^ | 5 (10.4%) | 4 (8.3%) | 4 (9%) | 2 (4.2%) | 1 (2.1%) | 1 (3%) |
| Grade 1^a^ | 16 (33.3%) | 18 (37.5%) | 17 (35%) | 9 (18.8%) | 9 (18.7%) | 9 (18%) |
| Grade 2^a^ | 7 (14.6%) | 8 (16.7%) | 7 (15%) | 12 (25.0%) | 14 (29.2%) | 13 (27%) |
| Grade 3^a^ | 20 (41.7%) | 18 (37.5%) | 19 (39%) | 25 (52.0%) | 24 (50.0%) | 24 (51%) |
| Increase | - | - | - | 17 (35.4%) | 20 (42.6%) | 18 (39%) |
| Decrease | - | - | - | 7 (14.9%) | 6 (12.8%) | 6 (13%) |
| No Change | - | - | - | 23 (48.9%) | 21 (44.7%) | 22 (46%) |

^a^Necrosis grade definitions: grade 0 = absent necrosis; grade 1 = <25% necrosis; grade 2 = 25–50% necrosis; grade 3 = >50% necrosis.

**Table S2.** Comparison of pre-treatment ADC values and treatment-related ADC changes ^a^ (absolute and percentage) between patients with localized and metastatic disease (Mann–Whitney test with FDR-adjusted *P* values)

| Variable | Localized disease (N=40) | | Metastatic disease (N=10) | | *P*value |
| --- | --- | --- | --- | --- | --- |
|  | **Mean(S.D.)** | **Median**  **[Q1;Q3]** | **Mean**  **(S.D.)** | **Median**  **[Q1;Q3]** |  |
| ADCmin^b^ | 791 (398) | 710 [573;938] | 1191 (524) | 1093 [789;1524] | 0.062 |
| ADCmean^b^ | 1203 (453) | 1119 [910;1447] | 1611 (603) | 1487 [1241;2147] | 0.085 |
| ADCmax^b^ | 1634 (545) | 1639 [1199;1949] | 2068 (748) | 2051 [1627;2708] | 0.103 |
| ADCsoft^b^ | **1260 (504)** | **1168 [955;1536]** | **1878 (635)** | **2152 [1450;2286]** | **0.028** |
| Δ ADCmin^b^ | 472 (596) | 254 [82.0;928] | 110 (505) | 188 [-251.88;390] | 0.139 |
| Δ ADCmean^b^ | 495 (647) | 494 [41.8;882] | 12.2 (524) | 23.8 [-247.88;366] | 0.062 |
| Δ ADCmax^b^ | 437 (810) | 356 [70.8;823] | -129.80 (665) | 48.8 [-322.38;252] | 0.066 |
| Δ ADCsoft^b^ | **598 (714)** | **578 [164;1085]** | **-307.15 (681)** | **-369.25 [-615.25;305]** | **0.018** |
| %Δ ADCmin^b^ | 98.4 (172) | 38.5 [9.97;129] | 13.2 (42.8) | 22.7 [-27.74;36.0] | 0.085 |
| %Δ ADCmean^b^ | **54.0 (62.4)** | **53.4 [4.15;94.7]** | **4.16 (29.3)** | **1.82 [-12.96;27.0]** | **0.036** |
| %Δ ADCmax^b^ | 39.3 (63.7) | 24.8 [2.59;70.6] | -1.14 (27.1) | 3.57 [-13.01;20.7] | 0.066 |
| %Δ ADCsoft^b^ | **79.1 (120)** | **47.6 [11.2;111]** | **-9.60 (38.9)** | **-16.54 [-41.14;16.8]** | **0.018** |

^a^ADC values and ΔADC values are expressed as ×10⁻⁶ mm²/s. %ΔADC values are expressed as percentages. ^b^ADCmin: minimum ADC, ADCmean: mean ADC, ADCmax: maximum ADC, ADCsoft: mean ADC of soft tissue component, ΔADC: absolute ADC variation, %ΔADC: relative ADC variation.

**Table S3** – Association of pre-treatment ADC values and treatment-related ADC changes^a^ (absolute and percentage) with time to relapse in multivariable Cox regression analysis (adjusted for age at diagnosis and clinical status).

| **Variable** | **HR** | **Lower 95% Limit** | **Upper 95% Limit** | *P* **value** |
| --- | --- | --- | --- | --- |
| **ADCmin**^b^ | 1.01 | 0.92 | 1.11 | 0.829 |
| ADCmean^b^ | 1.00 | 0.91 | 1.09 | 0.922 |
| ADCmax^b^ | 0.98 | 0.91 | 1.06 | 0.664 |
| ADCsoft^b^ | 1.00 | 0.92 | 1.08 | 0.959 |
| Δ ADCmin^b^ | 1.03 | 0.95 | 1.11 | 0.520 |
| %Δ ADCmin^b^ | 1.00 | 1.00 | 1.00 | 0.631 |
| Δ ADCmean^b^ | 1.00 | 0.92 | 1.08 | 0.930 |
| %Δ ADCmean^b^ | 1.00 | 0.99 | 1.01 | 0.521 |
| Δ ADCmax^b^ | 0.99 | 0.93 | 1.06 | 0.806 |
| %Δ ADCmax^b^ | 1.00 | 0.99 | 1.01 | 0.618 |
| Δ ADCsoft^b^ | 1.03 | 0.96 | 1.11 | 0.388 |
| %Δ ADCsoft^b^ | 1.00 | 1.00 | 1.01 | 0.136 |

^a^ADC values and ΔADC values are expressed as ×10⁻⁶ mm²/s. %ΔADC values are expressed as percentages. ^b^ADCmin: minimum ADC, ADCmean: mean ADC, ADCmax: maximum ADC, ADCsoft: mean ADC of soft tissue component, ΔADC: absolute ADC variation, %ΔADC: relative ADC variation.

**Table S4** – Association of pre-treatment ADC values and treatment-related ADC changes^a^ (absolute and percentage) with time to death in multivariable Cox regression analysis (adjusted for age at diagnosis and clinical status).

| **Variable** | **HR** | **Lower 95% Limit** | **Upper 95% Limit** | *P* **value** |
| --- | --- | --- | --- | --- |
| **ADCmin**^b^ | 1.01 | 0.92 | 1.11 | 0.829 |
| ADCmean^b^ | 1.00 | 0.91 | 1.09 | 0.922 |
| ADCmax^b^ | 0.98 | 0.91 | 1.06 | 0.664 |
| ADCsoft^b^ | 1.00 | 0.92 | 1.08 | 0.959 |
| Δ ADCmin^b^ | 1.03 | 0.95 | 1.11 | 0.520 |
| %Δ ADCmin^b^ | 1.00 | 1.00 | 1.00 | 0.631 |
| Δ ADCmean^b^ | 1.00 | 0.92 | 1.08 | 0.930 |
| %Δ ADCmean^b^ | 1.00 | 0.99 | 1.01 | 0.521 |
| Δ ADCmax^b^ | 0.99 | 0.93 | 1.06 | 0.806 |
| %Δ ADCmax^b^ | 1.00 | 0.99 | 1.01 | 0.618 |
| Δ ADCsoft^b^ | 1.03 | 0.96 | 1.11 | 0.388 |
| %Δ ADCsoft^b^ | 1.00 | 1.00 | 1.01 | 0.136 |

^a^ADC values and ΔADC values are expressed as ×10⁻⁶ mm²/s. %ΔADC values are expressed as percentages. ^b^ADCmin: minimum ADC, ADCmean: mean ADC, ADCmax: maximum ADC, ADCsoft: mean ADC of soft tissue component, ΔADC: absolute ADC variation, %ΔADC: relative ADC variation.

**Table S5.** Receiver operating characteristic (ROC) analysis of ADC parameter changes for relapse prediction

| Variable | AUC | Lower Limit | Upper Limit |
| --- | --- | --- | --- |
| ΔADCmin^b^ | 0.655 | 0.496 | 0.815 |
| ΔADCmean^b^ | **0.667** | **0.509** | **0.825** |
| ΔADCmax^b^ | 0.654 | 0.496 | 0.811 |
| ΔADCsoft^b^ | 0.635 | 0.466 | 0.804 |

ADC values and ΔADC values are expressed as ×10⁻⁶ mm²/s. ADCmin: minimum ADC, ADCmean: mean ADC, ADCmax: maximum ADC, ADCsoft: mean ADC of soft tissue component, ΔADC: absolute ADC variation, %ΔADC: relative ADC variation.
